# Signal transduction-related responses to phytohormones and environmental challenges in sugarcane

**DOI:** 10.1186/1471-2164-8-71

**Published:** 2007-03-13

**Authors:** Flávia R Rocha, Flávia S Papini-Terzi, Milton Y Nishiyama, Ricardo ZN Vêncio, Renato Vicentini, Rodrigo DC Duarte, Vicente E de Rosa, Fabiano Vinagre, Carla Barsalobres, Ane H Medeiros, Fabiana A Rodrigues, Eugênio C Ulian, Sônia M Zingaretti, João A Galbiatti, Raul S Almeida, Antonio VO Figueira, Adriana S Hemerly, Marcio C Silva-Filho, Marcelo Menossi, Gláucia M Souza

**Affiliations:** 1 Departamento de Bioquímica, Instituto de Química, Universidade de São Paulo, São Paulo, SP, Brazil; 2 BIOINFO-USP Núcleo de Pesquisas em Bioinformática, Universidade de São Paulo, São Paulo, SP, Brazil; 3 Centro de Biologia Molecular e Engenharia Genética, Universidade Estadual de Campinas, Campinas, SP, Brazil; 4 Instituto de Bioquímica Médica, Universidade Federal do Rio de Janeiro, UFRJ, Rio de Janeiro, RJ, Brazil; 5 Departamento de Genética, Escola Superior de Agricultura Luiz de Queiroz, ESALQ, Universidade de São Paulo, Piracicaba, SP, Brazil; 6 Centro de Tecnologia Canavieira, Piracicaba, São Paulo, SP, Brazil; 7 Departamento de Tecnologia, Faculdade de Ciências Agrárias e Veterinárias de Jaboticabal, Universidade Estadual Paulista, Jaboticabal, SP, Brazil; 8 Centro de Energia Nuclear na Agricultura (CENA), Universidade de São Paulo, Piracicaba, SP, Brazil

## Abstract

**Background:**

Sugarcane is an increasingly economically and environmentally important C4 grass, used for the production of sugar and bioethanol, a low-carbon emission fuel. Sugarcane originated from crosses of *Saccharum *species and is noted for its unique capacity to accumulate high amounts of sucrose in its stems. Environmental stresses limit enormously sugarcane productivity worldwide. To investigate transcriptome changes in response to environmental inputs that alter yield we used cDNA microarrays to profile expression of 1,545 genes in plants submitted to drought, phosphate starvation, herbivory and N_2_-fixing endophytic bacteria. We also investigated the response to phytohormones (abscisic acid and methyl jasmonate). The arrayed elements correspond mostly to genes involved in signal transduction, hormone biosynthesis, transcription factors, novel genes and genes corresponding to unknown proteins.

**Results:**

Adopting an outliers searching method 179 genes with strikingly different expression levels were identified as differentially expressed in at least one of the treatments analysed. Self Organizing Maps were used to cluster the expression profiles of 695 genes that showed a highly correlated expression pattern among replicates. The expression data for 22 genes was evaluated for 36 experimental data points by quantitative RT-PCR indicating a validation rate of 80.5% using three biological experimental replicates. The SUCAST Database was created that provides public access to the data described in this work, linked to tissue expression profiling and the SUCAST gene category and sequence analysis. The SUCAST database also includes a categorization of the sugarcane kinome based on a phylogenetic grouping that included 182 undefined kinases.

**Conclusion:**

An extensive study on the sugarcane transcriptome was performed. Sugarcane genes responsive to phytohormones and to challenges sugarcane commonly deals with in the field were identified. Additionally, the protein kinases were annotated based on a phylogenetic approach. The experimental design and statistical analysis applied proved robust to unravel genes associated with a diverse array of conditions attributing novel functions to previously unknown or undefined genes. The data consolidated in the SUCAST database resource can guide further studies and be useful for the development of improved sugarcane varieties.

## Background

Sugarcane is an increasingly economically attractive crop, used for the production of approximately 60% of the world's sugar and also of ethanol, a low-carbon emission fuel. Sugarcane varieties with improved tolerance to adverse environmental conditions are highly desirable. Unfavorable environmental factors are the major culprits of losses in agriculture and can reduce average productivity by 65% to 87% depending on the crop [1]. Crops better fit to withstand biotic and abiotic stresses have been selected by traditional genetic breeding programs but the slow pace in obtaining plants with the desirable traits limits the development of improved crop varieties. In this scenario, the use of molecular tools that enable gene-targeted modifications to achieve a phenotype of interest is highly promising.

Plants react to changes in the environment through an array of cellular responses that are activated by stress stimuli, leading to plant defense and/or adjustment to adverse conditions. Physiological changes elicited by external signals can be modulated by transcriptional regulation leading to the induction or repression of target genes. Many high throughput studies have been conducted to define gene expression changes in plants submitted to stress [2-5]. Such studies showed that signal transduction gene expression is altered in response to stress possibly leading to changes in growth and development and adjustment to environmental conditions. Few studies have been conducted to unravel sugarcane's responses to biotic and abiotic stresses or the role of phytohormones in these processes. Examples of these are those that evaluated changes in the sugarcane transcriptome induced by cold and methyl jasmonate treatment [6,7]. The aim of this work was to profile sugarcane gene expression under conditions that affect crop yield: drought, phosphate starvation, herbivory and endophytic bacteria interaction. Drought is a condition of special interest, not only for sugarcane, but also for other crops, since increasing water scarcity has been observed throughout the world. Plant irrigation currently accounts for approximately 65% of global freshwater use indicating that the development of plant varieties resistant to drought will be a necessity in the near future [8,9]. Plant responses to drought are complex, partially dependent on ABA signaling and dependent on the intensity and duration of the stimulus. The main responses include changes in ion fluxes, stomatal closing, production of osmoprotectants and alteration in plant growth patterns [9].

A significant portion of the arable land in tropical areas presents either limiting concentrations of essential nutrients or toxicity. Phosphorous (P), an essential macronutrient, is one of the most limiting nutrients for plant growth because of its low solubility and high sorption capacity in soil [10]. Plant roots acquire P as inorganic phosphate (Pi), although the concentration of Pi in the soil solution is often low (2 to 10 mM) [11]. The low availability of Pi in the acid soils of tropical and subtropical regions is a major limiting factor for crop production [12]. P constitutes around 0.2% of plants dry weight [13] and plays important roles in several biological processes, such as nucleic acid and phospholipid biosynthesis, energy metabolism, signal transduction and enzyme activity regulation.

Insect pests frequently challenge sugarcane productivity. The sugarcane borer *Diatraea saccharalis *is the major sugarcane pest in Brazil causing plant death due to apical bud death (dead heart) in plants of up to four months of age and damage to lateral bud development, aerial rooting, weight loss and stalk breakage in older plants. The attack also allows for infection by opportunistic fungi, which results in production loss for both the sugar and alcohol industries [[14] and references herein].

The sugarcane culture is highly benefited by the association with N_2_-fixing endophytic bacteria (*Herbaspirillum seropedicae/Herbaspirillum rubrisubalbicans *and *Gluconacetobacter diazotrophicus*). Unlike rhizobium/leguminosae symbiosis, where bacteria are restricted to nodules, *Herbaspirillum *spp. and *G. diazotrophicus *are endophytic, and colonize intercellular spaces and vascular tissues of most plant organs without causing damage to the host [15,16]. These bacteria promote plant growth possibly by nitrogen fixation and also by the production of plant hormones [17]. Despite the non-pathogenic aspects of this interaction, plants should limit bacterial growth inside their tissues, or the association can result in disease [18]. Little is known about the signaling mechanisms that are involved in the establishment of a beneficial association with the plant.

A study on the response of sugarcane plants to methyl jasmonate (MeJA) and abscisic acid (ABA) treatments is needed since the role of these phytohormones in biotic and abiotic stress responses is well characterized and could point us to the regulatory mechanisms behind the stress treatments of interest. Several evidences point to a complex signaling network triggered by the action of ABA, including cross-talk with other hormone response pathways [19]. Moreover, several genes that are induced by ABA also have their expression induced by drought and cold stress [20]. Protein kinases [21] and transcription factors [22] have been shown to mediate the signal transduction network of MeJA action. All MeJA actions seem to need a functional COI protein, involved in ubiquitin-mediated proteolysis [23]. cDNA arrays have been used to evaluate changes in gene expression in sugarcane leaves treated with MeJA [7]. Two transcriptional factors encoding a putative zinc finger protein, a heat shock factor protein, protein kinases, proteins with a role in secondary metabolism, protein synthesis, stress response and photosynthesis were found to be differentially expressed.

The genes studied in this work were identified by the SUCEST (Sugarcane EST) Project. The SUCEST Project [24] sequenced over 238,000 ESTs, which were grouped into over 43,000 SAS (Sugarcane Assembled Sequences) [25]. The SUCAST Project (Sugarcane Signal Transduction) [26,27] used BLAST searches, Pfam and SMART domain analysis to identify conserved signal transduction components such as receptors, adapters, G-proteins, small GTPases, members of the two-component relay system, nucleotide cyclases, protein kinases, protein phosphatases, elements of the ubiquitination machinery and transcription factors. In addition, SAS that might be involved in processes triggered by stress and pathogens or play a role in growth and development were also catalogued. The combined analysis of the sugarcane EST data bank, by means of an in depth annotation and gene architecture analysis, generated the SUCAST catalogue with over 3,500 members including around 100 SAS for hormone biosynthesis and around 600 SAS with no similarities to known proteins, which were selected due to our interest in associating function to new genes. These elements represent 5% of the total SAS from the current SUCEST dataset.

To define the expression pattern of these genes in the various sugarcane tissues cDNA microarrays with 1,280 distinct elements were constructed. A total of 217 genes were found to be differentially expressed when leaf, inflorescence, root, internode and lateral bud tissues were compared [27]. For this work, a new array was designed with 1,228 elements in common with the array used in the previous study [27] plus an additional 317 elements including 229 representatives of the sugarcane kinome. Overall, 50% of the SAS catalogued in each SUCAST category are represented in the array that contains a total of 1,545 genes.

In the context of plant signal transduction, the role of protein kinases is remarkable. These proteins are responsible for the post-translational control of target proteins, acting as critical regulators of many signaling cascades. Moreover, many plant protein kinases act as receptors (named RLKs, from Receptor-Like Kinases) and participate in processes like disease resistance, growth, development, hormone perception and stress responses [28]. Many protein kinases remain uncharacterized, especially those corresponding to RLKs. Of 1,031 protein kinases previously catalogued by the SUCAST Project 39% could not be assigned to known categories based on BLAST searches and were annotated as undefined kinases. This work also reports the categorization of sugarcane protein kinases based on neighbor-joining (NJ) trees constructed from the alignment of the predicted catalytic domain. The association of an expression pattern to the categories generated by the phylogenetic analysis is useful in guiding studies on sugarcane kinases and other genes responsive to environmental and hormonal stimuli.

## Results

### Gene Expression Changes in Response to Biotic and Abiotic Stimuli

To identify genes regulated at the expression level by biotic and abiotic factors sugarcane plants were exposed to a variety of conditions that affect yield negatively (drought, phosphate deficiency, herbivory) or positively (endophytic bacteria interaction). Since a role for ABA and jasmonates has been observed in the regulation of plant stress responses in other plant systems [19,29-32], plants were also exposed to these phytohormones.

To obtain gene expression patterns and identify differentially expressed genes cDNA microarrays representing 1,545 genes were co-hybridized to fluorescently labeled probes generated from control and treated plants. The great majority of the genes were selected from the SUCAST Catalogue [26,27]. Some correspond to sugarcane metabolism genes indexed in the SUCAMET (Sugarcane Metabolism) Catalogue. Both catalogues can be found at [33]. The hybridizations were performed as shown in Table 1. Cultivar SP80-3280 was used for the ABA, MeJA, phosphate deficiency and herbivory experiments. Cultivar SP90-1638 was used for drought experiments and SP70-1143 for the endophytic bacteria interaction experiments. To define differential expression we used the outliers searching method [34].

A total of 179 genes were identified as differentially expressed in both biological replicates in at least one of the treatments. Of these, twenty-nine were found differentially expressed in two or more treatments. Most of these (18) were responsive both to drought and phytohormones in agreement with the known role of ABA and MeJA in drought responses as discussed in the next section.

Additional file 1: Table 1 lists the differential expression (induction or repression) observed for each SAS in each treatment as well as the corresponding SUCAST categories. All additional files may be found at [35]. For reference, the table also includes the tissue expression profile for these genes in flowers, lateral buds, leaves, roots, immature and mature internodes (1^st ^and 4^th ^internodes, respectively) as published previously [27]. The log_2 _ratio (M) values for the valid elements represented in our array for all experiments are shown in additional file 2: Table 2.

Drought elicited changes were most apparent in the late experimental data points (72 h and 120 h) as opposed to the first data point (24 h): 88% of drought-responsive genes were detected as differentially expressed exclusively after 72 h and/or 120 h of water deprivation. Conversely, the majority (78%) of the genes regulated by phosphate deficiency were detected as differentially expressed in the early data point (6 h). For the phytohormone treatments, differential expression was found throughout the experimental time-course.

**Figure 1:**
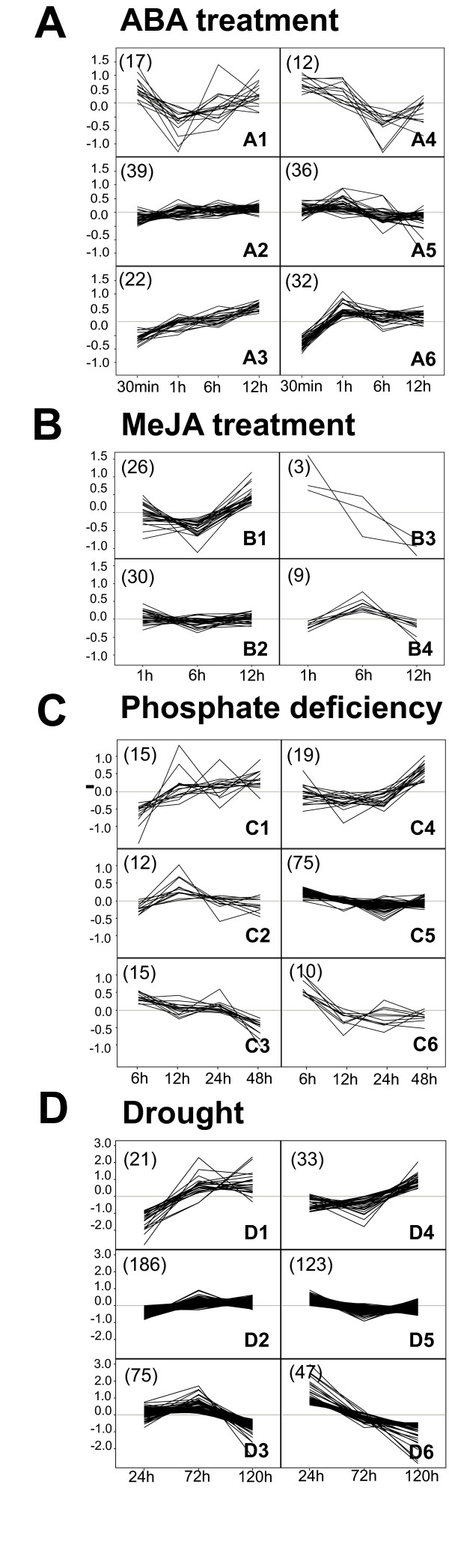
SOM analysis for (A) ABA and (B) MeJA treatments, (C) phosphate deficiency and (D) drought. Genes were selected based on a correlation coefficient of 0.7 or higher in the expression pattern obtained for the two biological replicates. The values of the median intensity ratios for each biological replicate were mean-centered and the average values were used as input for the SOM clustering. The geometry was chosen based on a PCA Analysis. The graphs present the average of the normalized log_2 _ratio (M) value between the replicates (y axis) plotted against the time course (x axis). The components of the SOM groups obtained are available in their totality as additional files (additional file 4: Table 4, additional file 5: Table 5, additional file 6: Table 6 and additional file 7: Table 7). The number in brackets indicates the number of SAS in each group.

In addition to the analysis of differential expression using the outliers searching method, the SOM algorithm [36] was used to cluster the expression data for phytohormone treatments, phosphate starvation and drought. Gene expression profiles were compared between the two biological replicates. Profiles with a correlation coefficient of 0.7 or higher were identified for 158 genes in response to ABA treatment, 68 in response to MeJA treatment, 146 for phosphate deficiency and 485 for drought. The clusters obtained are partially shown in Figure 1 and additional files 1 and 3. The components of the SOM groups are available as additional files (see additional file 4: Table 4, additional file 5: Table 5, additional file 6: Table 6 and additional file 7: Table 7). Many of the genes included in a SOM group showing evident induction or repression patterns were not detected as being differentially expressed according to the outliers searching method. While the outliers searching method is based on criteria that take into account the intensity-dependent effect on the ratio values and data reproducibility, the clustering analysis allows for the visualization of the expression pattern along the entire time course. For this reason, both analysis were taken into account when defining sugarcane genes responsive to these treatments.

### The Sugarcane Kinome

Among the differentially expressed genes defined by the outliers search method and the SOM groups we found 185 SAS belonging to the sugarcane kinome (additional file 3: Table 3). Since 39% of SUCAST protein kinases could not be classified based on BLAST similarities and domain analysis we used a phylogenetic approach based on the analysis of *Arabidopsis thaliana *protein kinases [37] to annotate the sugarcane kinome.

**Figure 2:**
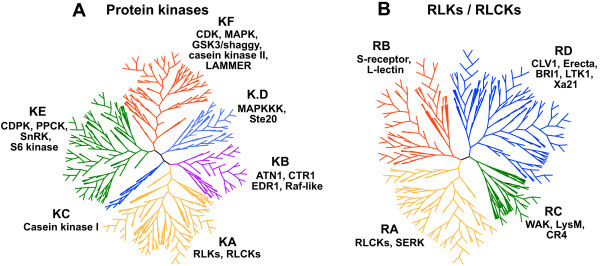
Phylogenetic analysis of sugarcane protein kinases (A) and RLKs/RLCKs (B). The predicted pkinase domains were aligned and used to construct a distance tree with the NJ algorithm. Only some of the main representatives of the RLK/RLCK category were included in the tree constructed for protein kinases (A). Driver sequences from other organisms were also included in the analysis. The main components of each group are: KA: RLKs and RLCKs; KB: ATN1, CTR1, EDR1, Raf-like; KC: casein kinase I; KD: MAPKKK, Ste20; KE: CDPK, PPCK, SnRK, S6 kinase; KF: CDK, MAPK, GSK3/shaggy, casein kinase II, LAMMER; RA: RLCKs, SERK; RB: S-receptor, L-lectin; RC: WAK, LysM, CR4; RD: CLV1, Erecta, BRI1, LTK1, Xa21. The complete trees indicating all SAS are available as additional files (additional file 6: Figure 1 and additional file 7: Figure 2).

Sugarcane protein kinases (277), RLKs (250) containing a putative *pkinase *domain and protein kinases from other organisms (156) were aligned using the neighbor-joining algorithm. The term RLCK (Receptor-like Cytoplasmic Kinase) was defined by [37] and refers to protein kinases that, in spite of having a catalytic domain very similar to the ones found for RLKs, apparently constitute cytoplasmic kinases. As it is known that RLKs/RLCKs form a monophyletic gene family with respect to other eukaryotic kinase families [37] we opted to first construct a NJ tree for sugarcane protein kinases, including only some representatives of the RLKs/RLCKs category, and then to obtain a NJ tree for RLKs/RLCKs members. A summarized view of the NJ trees obtained is depicted in Figure 2. A complete view of the neighbor-joining (NJ) trees is shown in additional file 8: Figure 1 and additional file 9: Figure 2. Six major groups were defined for protein kinases (KA, KB, KC, KD, KE and KF) with group KA comprising the RLKs and RLCKs representatives. Four groups were obtained (RA, RB, RC and RD) for the RLKs/RLCKs.

We observed that some SAS with BLAST best hits similar to undefined protein kinases and with no predicted transmembrane regions grouped with receptors and RLCKs in the phylogenetic analysis. For this reason, we classified these sequences as putative RLCKs instead of undefined protein kinases. In fact, some of these sequences may represent novel types of RLCKs not yet characterized. On the other hand, some of them may also be receptor-like kinases with incomplete cDNAs, lacking the extracellular domain and transmembrane region that would indicate they are receptors.

All sugarcane protein kinases were classified in the SUCAST database with the prefix cane followed by its annotation and a continuous numeration. Each undefined protein kinase or RLK received a new classification, based on the phylogenetic group and family to which it belongs. All families constituted entirely by sugarcane undefined protein kinases, RLKs or RLCKs, and supported by a bootstrap value superior to 50% received a specific nomenclature, as well as the SAS included within these families (additional file 8: Figure 1 and additional file 9: Figure 2). With this criterion, it was possible to define 6 families constituted entirely by undefined protein kinases and 33 families constituted by undefined RLKs/RLCKs.

The phylogenetic analysis allowed for the classification of 32 undefined protein kinases (additional file 8: Figure 1). Group KB contains 16 of them. Thirty-four undefined RLCKs and 117 undefined RLKs were included within the RLKs/RLCKs tree (additional file 9: Figure 2). Group RA contains most of the undefined RLCKs (76%) and group RD, the majority of the undefined RLKs (57%).

Among the 1,031 sugarcane protein kinases catalogued, 475 were represented in our array. Additional file 3: Table 3 shows expression data for all sugarcane protein kinases that were found as differentially expressed based on the outliers searching method (29) or SOM analysis (174).

### Validation of microarray data by real-time PCR

Twenty-two genes were selected to have their expression data validated by quantitative real-time PCR. The primers designed for these genes and the statistical analysis (probability Pr(sample>reference) and Pr(sample < reference) for up- and down-regulated genes, respectively) of the data are shown in additional file 10: Table 8. The expression profile along the whole time-course was analysed by real-time PCR for phytohormone treatment samples. For other treatments, reactions were carried out only for the experimental point(s) in which the gene was detected as being differentially expressed.

As reference genes for normalization we used a polyubiquitin gene for the ABA treatment and drought data, a GAPDH gene for the MeJA treatment and herbivory data, a 25S rRNA gene for endophytic inoculation and a 14-3-3 gene for the phosphate starvation data. The different references were selected for their unaltered expression in each of the treatments. Curves (log fluorescence × cycles) obtained at different experimental points and the respective SAS expression profiles in the M × S space for each particular experiment were evaluated. The polyubiquitin and 14-3-3 genes were previously used as a reference for the validation of expression levels in different sugarcane tissues [27]. The GAPDH and 25S rRNA genes were described as good references for sugarcane tissues and genotypes [38]. The 25S rRNA primers used were 25S rRNA1F and 25S rRNA1R [38].

**Figure 3:**
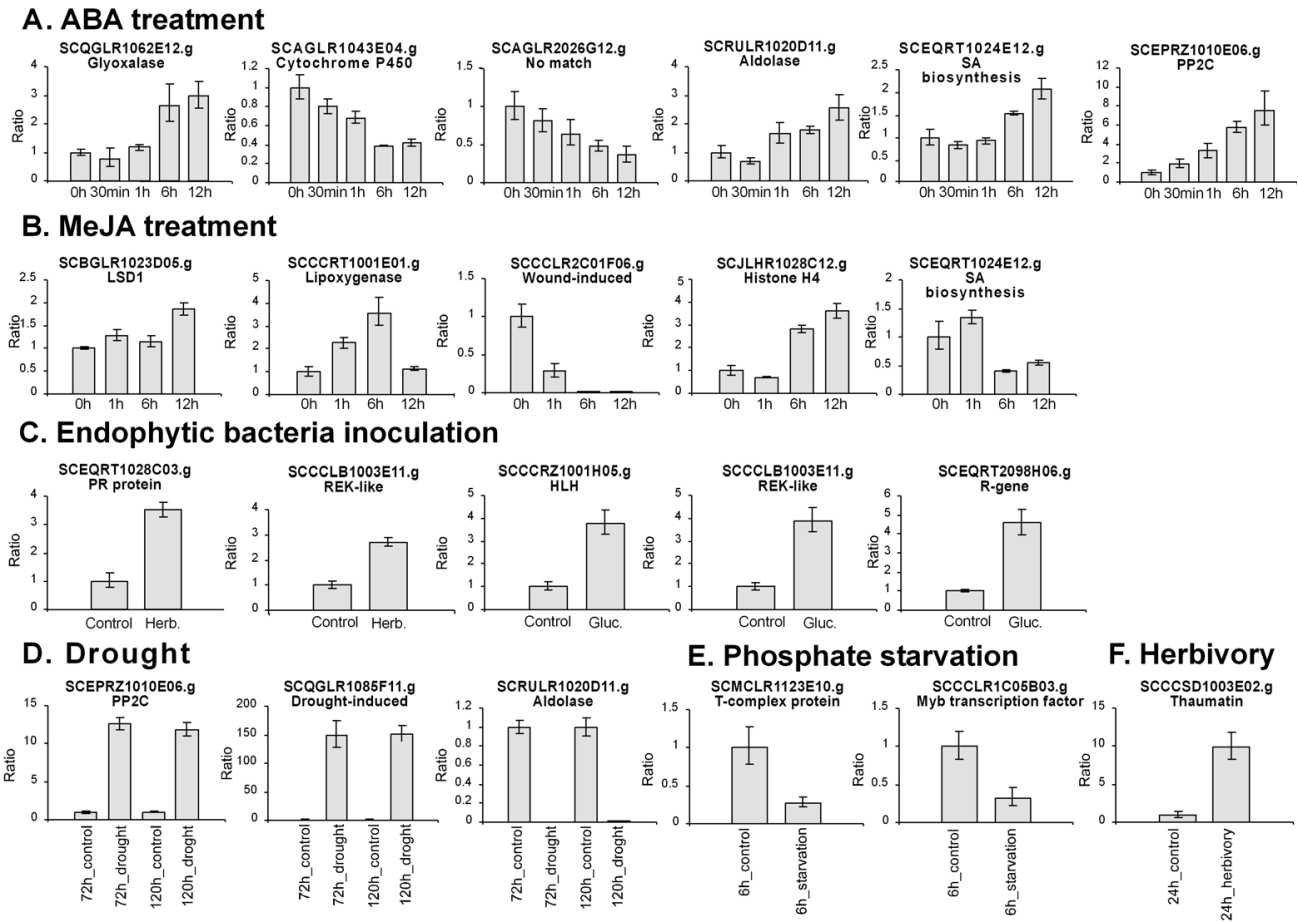
Validation of microarray results by quantitative PCR analysis. The y axis refers to the relative expression ratio between treated samples versus the control (untreated sample). (A) and (B) Real-time PCR results for ABA and MeJA treatments, respectively. The ratios were calculated in relation to the sample from untreated plants (0h). Transcript levels of the selected genes were profiled throughout the treatment time course. Also shown are the results for plant-endophytic bacteria association (C), drought (D), phosphate starvation (E) and herbivory (F). For these treatments, the real-time PCR reactions were carried out exclusively for the experimental point(s) in which the gene was considered differentially expressed. Only validated results are shown here. The RNA samples used in the real-time PCR experiments are from a third biological sample. All reactions were carried out in parallel and each reaction was performed in triplicates. Error bars were calculated as described previously [135]. Herb. = sample from plants inoculated with *Herbaspirillum seropedicae *and *Herbaspirillum rubrisubalbicans*; Gluc. = sample from plants inoculated with *Gluconacetobacter diazotrophicus*. The transcript levels for the reference genes were verified to not vary in response to the treatments. The reference genes used encode a polyubiquitin gene (SCCCST2001G02.g [CA179923]) for the ABA and drought dataset, a GAPDH for the MeJA and herbivory dataset (retrieved from [38]), a 25S rRNA for the endophytic inoculation (retrieved from [38]) and a 14-3-3 gene (SCCCLR1048F12.g [CA119519]) for phosphate starvation

A total of 36 results of differential expression were evaluated (additional file 10: Table 8). Of these, 80.5% had a profile in real-time PCR assays consistent with the one observed in the microarray experiments (probability value of 0.99 or higher). Validated real-time PCR results are depicted in Figure 3. It is important to emphasize that the RNA samples used in the real-time PCR experiments derived from a third biological sample and that the principles of real-time PCR techniques are different from the ones applied in microarray experiments. The conflicting results may correspond to biological variations in the third biological replica or even to technical limitations of the microarray method. Nevertheless, our analysis and statistical methods were efficient in evaluating differentially expressed genes, yielding only a minor percentage of unconfirmed expression data.

### The SUCAST Database

A database containing all the SAS catalogued in the SUCAST Project and their respective expression data was built and is available at The SUCEST-FUN Database web site [33]. The SUCEST-FUN database includes the expression data associated to stress responses and environmental stimuli and the expression profile of SUCAST SAS in six different sugarcane tissues [27]. It also includes the SUCAMET categories of sugarcane metabolism genes.

The SUCAST databank integrates the sequence data and analysis from the SUCEST Project [25], the categorization and tissue gene expression of signal transduction genes [27] with the kinome analysis and gene expression data in response to different treatments as pointed out by the outliers searching method, SOM and quantitative PCR analysis (this work).

The SUCAST system consists of a client web interface and a server back end. The database was constructed using the MySQL database server [39]. The scripts were written in Perl [40] and R statistical language [41]. Through the web interface, the SUCAST database can be easily queried to find each SAS and its associated information. For each SAS it is possible to retrieve the consensus sequence of the SAS and the alignment of its corresponding reads, according to the clusterization of SUCEST reads [25].

Besides expression data, the SUCAST database also provides, for each SAS queried, information regarding annotation, results of the blasts against the GenBank nr and the NCBI GEO databases and comparisons with the Gene Ontology database [42]. As of the time of submission 1,083 SAS showed similarity against sequences in NCBI GEO database (using BLASTN tool, cutoff Evalue of 10^-5^). Comparison of these SAS with the Gene Ontology database (using BLASTX tool, cutoff Evalue of e^-5^) revealed significant matching with proteins for 3,030 SAS.

Over 3,600 SAS are compiled in the SUCAST database. It is possible to retrieve the predicted protein sequence encoded by the consensus sequence as well as information on conserved protein domains predicted by the Pfam [43] databank. The SUCAST elements are distributed into 46 main categories subdivided into 1,678 subcategories. The database also includes 405 SUCAST SAS that present no matches with existing protein sequences in the GenBank nr database (using BLASTX tool, cutoff E value of 10^-5^).

## Discussion

### Identification of sugarcane genes responsive to biotic and abiotic stimuli

In an effort to associate gene expression changes to environmental factors that may affect sugarcane yield we profiled the expression of 1,545 sugarcane genes in response to drought, phosphate deficiency, herbivory and endophytic bacteria. We also analysed the responses of plants to ABA and MeJA treatments. The 1,545 genes selected are representative of all categories found in the SUCAST Catalogue. The outliers searching method has proved to be a robust way of differential expression analysis that considers the overall dispersion of signal intensity and provides reliable gene expression differences. A complementary approach useful to highlight the patterns observed and to include data points where differential expression was not striking was the selection of patterns highly correlated among biological replicates and their grouping by the SOM algorithm. This approach increased the number of genes analysed and confirmed the differences observed through the use of the outliers searching method. For instance, there were three histone genes (two H4 histone and one H2B histone) in SOM group B1 of genes up-regulated by MeJA. Only one of the H4 histone genes was detected as being up-regulated by MeJA exposure (after 12 h) according to the outliers searching method. This exemplifies the usefulness of the SOM analysis in addition to the outliers analysis of differentially expressed genes, revealing two additional histones induced by MeJA. Probably, the values of log_2 _ratio (M) for these SAS were not enough to surpass the intensity-dependent cutoff levels that indicated differential expression, but the SOM analysis revealed that these SAS apparently were responsive to the MeJA treatment.

From the 179 genes detected as differentially expressed, 113 were also included in at least one of the SOM clustering analysis performed (additional file 1: Table 1). Some had their expression patterns evaluated by real-time PCR along the entire time course of phytohormone treatment (SCBGLR1023D05.g [CA117725], SCJLHR1028C12.g [CA106117], SCEQRT1024E12.g [CA132523], SCQGLR1062E12.g [CA124203] and SCRULR1020D11.g [CA125940]) and presented consistent results.

### Analysis of sugarcane genes responsive to phytohormones and environmental challenges

Tissue expression data was obtained for most genes of the array in a previous study where samples extracted from six different sugarcane tissues were hybridized against a common reference sample [27]. These studies indicated 217 genes differentially expressed in at least one of the tissues tested and 153 genes evenly expressed in all tissues. Among the 179 differentially expressed genes detected in the present study, 70 correspond to tissue-enriched genes. Most of them (19) are leaf-enriched, but there were also genes that were expressed preferentially in sugarcane roots (12) and internodes (11). While these are interesting observations, it is important to emphasize that they should be considered with caution, since the experiments reported here used plantlets cultivated under growth chamber or greenhouse conditions while in our previous studies most of the samples were collected from 12- or 14-month-old plants cultivated in the field.

Water deprivation was the condition that elicited the majority of gene expression changes. Fifty-two percent of the 179 differentially expressed genes were responsive to drought. Many studies have reported the identification of genes regulated by drought and altered expression of transcription factors. Specific recognition sequences for some categories of transcription factors were detected in the promoter of drought-responsive genes, as is the case of MYC and MYB recognition sequences and the W-box for WRKY transcription factors [44,45]. We observed the induction of one MYB and two WRKY transcription factors in response to drought (group D4).

Many of the genes responsive to drought are similar to genes that in other systems have been shown to transduce additional stress signals including cold. The cold and drought signaling pathways present a high degree of overlap and many of the responses are mediated by ABA [46]. Four SAS encoding low temperature induced (LTI) proteins were up-regulated in response to lack of water. Two of these SAS (SCUTST3084F06.g [CA186860] and SCACCL6008H06.g [CA096029], group D1, Figure 1) are grouped with genes induced by water deprivation. Many genes co-regulated in response to drought and cold in *Arabidopsis *[47] have a DRE (Dehydration-Responsive Element) motif or DRE-related motifs in their promoters. A transcription factor (SCBGLR1002A09.g [CA117666]) from the AP2 family and homologous to rice DREB2 (DRE binding factor 2) [AAP70033] was induced after 72 h of watering suppression and may represent an important transcription factor for the regulation of sugarcane drought responsive genes. The overexpression of a constitutive active form of DREB2A [O82132] in *Arabidopsis thaliana *led to the development of transgenic plants more tolerant to drought [48]. Hence, the manipulation of DREB2 levels in sugarcane may represent a way of obtaining new varieties with increased resistance to water deficit. Genes induced by ABA and drought include two delta-12 oleate desaturase (SCCCLR1C03G01.g [CA189695] and SCVPST1061G05.g [CA179715]), one S-adenosylmethionine decarboxylase (SCCCLR1C05G07.g [CA189868]) and one PP2C-like protein phosphatase (SCEPRZ1010E06.g [CA147516]) homologous to *Arabidopsis *protein phosphatases ABI1 and ABI2. The protein phosphatases ABI1 and ABI2 are responsive to ABA and regulate a range of physiological responses, including stomatal closure, which minimizes the transpirational water loss [49,50]. S-adenosylmethionine decarboxylases participate in the polyamine biosynthetic pathway, which is modulated in response to abiotic stresses [51]. SCCCLR1C05G07.g [CA189868] is homologous to the S-adenosylmethionine decarboxylase *SAMDC1 *[AF067194], from rice, known to accumulate in response to salinity and drought, probably through ABA-dependent pathways and with an expression positively correlated with salt tolerance [52]. The regulation of fatty acid desaturases (FAD2) may be related to changes in the degree of fatty acid desaturation in response to environmental stresses. FAD3, FAD7 and FAD8 desaturases expression is directly related to drought tolerance. A role of an omega-3 fatty acid desaturase in drought tolerance was reported in tobacco through overexpression [53] and gene silencing [54] studies. Additionally, it was demonstrated that the reduction in trienoic fatty acid levels by the antisense expression of the *fad7 *[D26019] gene seems to affect the ABF (ABA responsive elements Binding Factor)-dependent gene expression showing a relationship between desaturases levels and ABA-signaling pathways [54]. Thus, the regulation of the sugarcane delta-12 oleate desaturases by ABA and drought may indicate that these genes are induced by drought and that this induction alters ABA signaling pathways.

A total of 31 differentially expressed genes were found in plants treated with ABA. Fifty-eight percent of these are exclusively regulated after 12 h of treatment with this hormone, indicating the existence of early and late response genes in the time course of our experiment. Among the ABA-responsive genes, we could observe the induction of a gene (SCCCLR1C07B07.g [CA189990]) encoding a glycine-rich protein with a predicted RNA recognition motif. The function of this class of proteins is not clear [55,56] but it is known that some of them play an important role in RNA turnover [57]. A *Sorghum bicolor *gene [AF310215] similar to SCCCLR1C07B07.g [CA189990] was induced by ABA treatment, light and salinity [58]. The maize MA16 protein is also an example of an RNA-binding protein induced by ABA [19,59].

Several regulators of ABA signaling pathways are described and characterized [19]. Among these, the ROP10 small GTPase [NP_566897] from the Rab family in *Arabidopsis *was implicated in the down-regulation of the ABA signal transduction pathway [60]. Our analysis revealed two GTPases similar to Rab11 (SCACCL6006D08.g [CA095849] and SCJFRT1059D05.g [CA134244]) induced after exposure to this hormone. The OsRab7 GTPase from rice [AAO67728] [61] and the Rab2 GTPase [AAD30658] from *Sporobolus stapfianus *[62] were also described as regulated by ABA. These observations point to an involvement of different Rab GTPases in the cellular responses activated by this hormone.

Among the genes up-regulated by MeJA treatment there were two H4 histones and one H2B histone genes in SOM group B1. The H4 histone SCCCLR2002G09.g [CA127138] was also identified as up-regulated after 12 h of MeJA exposure by the outliers searching method. It has been reported that jasmonates may regulate gene expression by interfering with histone acetylation and deacetylation since COI1 [O04197], an F-box protein required for jasmonates responses was able to target an *Arabidopsis *histone deacetylase to proteolysis [23]. Furthermore, Kim and colleagues [63] observed the induction of histones CaH2B [AF038386] and CaH4 [AF038387] from *Capsicum annuum *by MeJA. The regulation of histone transcript levels in sugarcane points towards chromatin remodeling as a possible event activated by jasmonates which may represent an important mechanism through which jasmonates regulate the expression of target genes.

A comparison of sugarcane and rice [64] ABA responsive genes indicates that a fructose-bisphosphate aldolase (SCRULR1020D11.g [CA125940]), a glyoxalase (SCQGLR1062E12.g [CA124203]) and a RUBISCO gene (SCCCLR1001E04.g [CA116155]) are induced by ABA in both grasses. The sugarcane fructose-bisphosphate aldolase induced by ABA is similar to the *NpAldP1 *[AB027001] gene expressed in *Nicotiana paniculata *leaves and repressed in response to saline stress [65]. The sugarcane aldolase gene was also regulated by drought and MeJA treatments (additional file 1: Table 1, Figure 1 groups A3 and D6) and according to our previous work [27] this SAS is enriched in sugarcane leaves. This suggests a potential role of this gene in signaling pathways specific of this organ. Another SAS encoding an aldolase seems to be slightly induced by ABA treatment (Figure 1, group A2). The analysis of transcripts levels for genes of the glycolytic and fermentation pathways in rice roots and shoots indicated the induction of an aldolase gene in response to saline stress [66]. In another work, an *Arabidopsis *aldolase gene was repressed by ABA [20]. A wealth of evidence has accumulated throughout the years revealing important interactions between sugar- and phytohormone pathways [67]. Additionally, ABA is implicated in the regulation of sugar transport and metabolism. The regulation of glycolytic enzymes by stressful conditions and the consequences of this regulation are particularly interesting for sugarcane, since these findings may indicate a relationship between sucrose accumulation and responses to stresses.

The identification of differential expression for genes with no homologs in the public databases ("no matches") as well as of SAS coding for unknown proteins is particularly valuable for the identification of their putative roles. We obtained 28 "no matches" differentially expressed in at least one of the experiments analysed. Of these, 13 are regulated by drought, 7 by inoculation with *Herbaspirillum*, 5 by ABA treatment, 4 by MeJA treatment, 3 by herbivory and 3 by phosphate starvation. Four of these are leaf-enriched genes. One of them was induced by both inoculation with N_2_-fixing endophytic bacteria and exposure to insect attack. This may indicate a possible role for this gene in general mechanisms of defense against biotic stimuli, including endophytic recognition and the activation of defense responses until the establishment of an efficient association. It is also interesting to point out that five of these 28 no matches genes do not present a predicted coding region and may represent non-coding transcripts. Recent studies have established important roles for some plant microRNAs in the regulation of processes like development, response to pathogens and hormone signaling [68]. Among the SUCEST sequences, 239 non-coding no matches were identified. Through the present studies we see an indication that five of them may have a role in stress responses since drought regulated three of them, methyl jasmonate treatment regulated one and inoculation with *Herbaspirillum *spp regulated another.

Phosphate starvation altered the expression of 14 genes. The majority of them (11) showed decreased levels after 6 hours of starvation. Expression data indicates that during this early phase of the stress response an alteration in protein N-glycosylation may occur, as can be inferred from the repression of a gene coding for an N-acetylglucosamine-1-phosphate transferase (SCRUFL1112F04.b [CA249652]). The decreased expression of two genes coding for thioredoxins (SCCCLR2001H09.g [CA127047] and SCJFLR1073B06.g [CA122039]) indicates there are changes in the redox state of sugarcane roots in response to phosphate starvation, since these enzymes are important regulators of the intracellular redox status [69]. A gene similar to MYB transcription factors had transcript levels reduced. Several members of this gene family show distinct expression profiles in response to phosphate starvation in *Arabidopsis*, some of them being up-regulated and some down-regulated [70]. It is worth to note that the promoter region of the oat homolog of this gene (*MybHv1*) [X70879] has been characterized and shown to be active only in the root apex [71]. Alterations in the expression of root apex enriched genes could be related to the morphological changes observed in the root system in response to low levels of P.

Even though the plant-endophytic association is advantageous for both organisms, it is believed that sugarcane plants recognize these microorganisms and activate defense responses until the establishment of an efficient association [72]. In agreement with this, four R-genes were found among the genes responsive to the endophytic association. Plant disease resistance (R) genes mediate specific recognition of pathogens via perception of avirulence (avr) gene products [73]. Two of them were induced by both associations under study (sugarcane-*Herbaspirillum *spp and sugarcane-*Gluconacetobacter diazotrophicus*). The inoculation with *Gluconacetobacter *also led to the induction of a salicylic acid biosynthesis gene. The phytohormone salicylic acid accumulates in plant tissues in response to pathogen attack and is essential for the induction of systemic acquired resistance and for some responses mediated by resistance genes [74-76].

A PP2C (SCJLRZ3077G10.g [CA160745]) was up-regulated in plants inoculated with *Gluconacetobacter*. Also, expression of five transcription factors was altered when the plants were cultivated in association with endophytic bacteria. Among these, there were two zinc-finger transcription factors (SCEQRT1033F01.g [CA133313] and SCEZST3147A10.g [CA182656]), one of which was up-regulated by inoculation with either *Gluconacetobacter *or *Herbaspirillum*. In agreement with our data, a possible role for phosphatases and zinc-finger transcription factors in response to endophytic bacteria has also been pointed out by the *in silico *analysis of the SUCEST Project libraries, that identified SAS corresponding to these categories exclusively or preferentially expressed in the SUCEST cDNA libraries constructed from plants inoculated with *Gluconacetobacter *and *Herbaspirillum *[77].

Expression data for the herbivory experiment points to the strong induction of a pathogenesis-related protein similar to a thaumatin after 24 h of the onset of this stress. This is not surprising, since it is known that proteins from this category are important for plant defense mechanisms and may present antifungal action, endo-β1,3-glucanase activity and trypsin or a-amylase inhibitory activity [78,79]. Further characterization of this sugarcane thaumatin-like protein should be carried out in order to define its activity and the defense mechanism that this protein may confer against the sugarcane stalk borer.

### Sugarcane-responsive genes related to hormone biosynthesis and signaling

Phytohormone signaling pathways exhibit a wide degree of cross-talk among their components creating a complex network of overlapping signaling [80,81]. Interactions among phytohormone signaling pathways are highly complex and the features of these interactions are time and space dependent. Although we are only beginning to outline signaling cross-talks in sugarcane, the analysis of the expression profiles of the differentially expressed genes obtained (additional file 1: Table 1) as well as the groups obtained in the clustering analysis (Figure 1 and additional files 4, 5, 6, 7) uncover some aspects of these interactions. Auxin signal transduction pathways appear to be activated in response to several of the treatments studied. ABA treatment elicited an antagonistic response between the ABA and auxin pathways. A gene (SCCCCL3002B05.b [CA093260]) coding for a protein similar to the auxin responsive protein GH3 [82] was found repressed by ABA (group A4). Furthermore, a gene (SCCCLR2002F08.g [CA127125]) coding for a protein with a predicted auxin repressed domain found in dormancy-associated- and auxin-repressed proteins [83] was up-regulated by this hormone (group A6). It has been shown that ABA and auxin interact antagonistically to regulate stomatal aperture [84] and the interaction between auxin and ABA signaling pathways has been demonstrated by the dual specificity of the ABI3 transcription factor, which is able to bind sequences upstream of ABA and auxin responsive genes. In the presence of ABA, ABI3 binds to the GH3 like promoter sequences and inhibits the auxin-mediated induction [85].

Auxins have been implicated in several aspects of the drought response including proline accumulation [86], rhizogenesis [87] and indole 3-butyric acid increases [88]. Our data also indicate auxin signaling in response to drought. We observed the induction of genes encoding auxin biosynthesis enzymes (nitrilases) after 72 h of water deprivation and the down-regulation of transcription factors from the Aux/IAA category after 120 h of drought. These transcription factors work by inhibiting auxin signaling and are rapidly induced by auxin exposure [89,90]. However, Aux/IAA accumulation is subject to negative feedback, since auxins target Aux/IAA for degradation by the 26S proteasome [90]. Groups D1 and D2 contain two nitrilases and one auxin-binding protein (ABP). Additionally, one auxin response factor is induced by drought (group D4) and four AUX/IAA transcription factors are modulated (groups D2, D3 and D6).

Phosphate starvation leads to alterations in root architecture, resulting in increased soil exploration and phosphate acquisition. In this process of morphological adaptation, auxins and other phytohormones play important roles in root elongation and lateral root development [91]. López-Bucio and colleagues [92] showed that phosphate deprivation increases auxin sensitivity in *Arabidopsis*, what may explain the increased number of lateral roots observed when the plant is under nutritional stress. In agreement with this observation, the auxin-repressed protein found in group A6 (SCCCLR2002F08.g [CA127125]) was repressed after 6 h of phosphate starvation. This same SAS was also down-regulated by MeJA treatment what may indicate a possible synergism among MeJA and auxin pathways. Even though the majority of interactions between MeJA and auxins are antagonistics, there is evidence that these hormones may act synergistically at the post-transcriptional level [93]. It is hypothesized that, since COI1 and TIR1, components of SCF (SKP1, CDC53p, CUL1, F-box protein) complexes associated to jasmonate- and auxin-responses, respectively, are highly similar, these two signaling pathways may converge to the degradation of common target regulatory proteins [93].

Phosphate starvation also causes a reduction in the expression of a gene (SCEZLB1009A09.g [CA113117]) similar to BLE1 from rice. Rice plants where the gene *OsBle1 *[AB072977] was knocked-out showed reduced growth rates [94]. The repression of the sugarcane homolog of *OsBle1 *in the early phase of phosphate starvation could be an effort to restrain metabolism as occurs in *Arabidopsis *in response to low levels of the nutrient [70]. Another hormone signaling pathway that seems to be altered in response to phosphate starvation is the ethylene response pathway, since a gene for the EIL transcription factor (SCBGFL4052C11.g [CA221542]) is down-regulated after 6 h of starvation. The rice homolog of this protein, OsEIL1 [AAZ78349], acts as a positive regulator of the ethylene response and transgenic rice plants overexpressing OsEIL1 exhibit short root, coiled primary root, slightly short shoot phenotype and elevated response to exogenous ethylene [95]. The down-regulation of this transcription factor could be related to the changes in root architecture that occur in response to phosphate starvation.

### The Sugarcane Protein Kinases and RLKs

Detailed descriptions of yeast, *Drosophila*, *C.elegans *and human kinomes are available [96,97] as well as studies on plant kinases [28,37,98]. Since plant protein kinases and RLKs act as critical regulators of many signaling pathways, they represent important targets to modify pathways of interest.

Eight protein kinases were differentially expressed in response to plant inoculation with N_2_-fixing bacteria. Three of these are similar to proteins involved in calcium signaling, a calcium-dependent protein kinase (caneCDPK-9) and two calcineurin B-like interacting protein kinases (caneCIPK-18 and caneCIPK-22) of the SnRK3 subgroup of plant kinases [99,100]. Some reports have shown the role of calcium-dependent pathways in the processes of symbiosis and nodulation [101-104]. CDPKs may participate in pathogen defense signaling pathways, as seen in the tomato defense responses against the fungi *Cladosporium fulvum *[105]. We also observed the induction of a sugarcane gene similar to a GSK3/shaggy protein (caneGSK3-5) kinase by endophytic bacteria association. In plants, these kinases are associated to floral development, brassinosteroid signaling pathways and responses to stresses such as wounding and salinity [106]. Moreover, one PBS1-like protein kinase (canePBS1-4) had its transcripts increased in response to the association. The *Arabidopsis *PBS1 [NP_196820] protein recognizes avirulence factors from *Pseudomonas syringae *[107]. The transcriptional regulation of this gene in sugarcane inoculated with N_2_-fixing bacteria suggests a possible role for this protein in the recognition of these microorganisms.

The role of some receptors in the regulation of symbiosis has been described [108-110]. Our data indicates the induction of a putative receptor with predicted leucin-rich repeats (caneURLK-13) in plants inoculated with *Herbaspirillum *that may be regulating such an interaction. Recently, a sugarcane receptor (SHR5) [AAY67902] was shown to be repressed in plants associated with endophytic bacteria and the degree of this repression was directly related to the success of the sugarcane-endophytic bacteria association, indicating a participation of this receptor in signal transduction pathways involved in the establishment of plant-endophytic bacteria interaction [72].

We identified ten protein kinases differentially expressed in response to drought. Seven of them are similar to the SnRK family of proteins. Four were induced by this stress (caneOsmotic stress-activated protein kinase-2, caneCIPK-8, caneCIPK-13 and caneCIPK-14). Some SnRKs are recognized players in stress responses. SRK2C leads to improved drought tolerance when overexpressed in *Arabidopsis thaliana *[111]. Mutagenesis studies on OST1 [NP_567945], a kinase whose activity is induced by drought, led to guard-cell specific effects and ABA insensitivity [9,112]. Other works describing the function of this family of protein kinases in drought responses include a role in stomatal closure [113-115]. Furthermore, among the drought responses, the Ca^2+^-dependent SOS signaling pathway (which involves SOS2, a SnRK similar to the CIPKs) has an important role in regulating ion homeostasis [116]. Since *sos2 *[NM_122932] mutants are hypersensitive to saline stress [117] it will be interesting to complement our studies with a phenotypic evaluation of plants altered for caneCIPK-8, caneCIPK-13 or caneCIPK-14 genes to confirm a role for these kinases in drought responses.

Among the differentially expressed genes, six undefined kinases/RLKs (caneRLCK-AVI2, caneRLCK-DII3, canePK-BIII3, caneRLK-AX1, caneRLK-AX2 and caneRLK-C5) were regulated by drought, inoculation with *Herbaspirillum *spp. and/or phytohormone treatments and 64 undefined kinases/RLKs were selected for the SOM clustering analysis. Six protein kinases that grouped within the same phylogenetic family in group KE have very similar catalytic domains, with an insertion of around 80 aminoacids between subdomains VII and VIII, as defined by Hanks *et al*. [118-120]. These proteins are similar to the protein kinase G11A from rice [AAA33905] [121]. One of these SAS (caneG11A kinase-2) was included in SOM group A2 and two of them (caneG11A kinase-3 and caneG11A kinase-5), in group D2, with an apparent expression profile of induction by ABA or drought, respectively. It may be of interest to evaluate potential targets of these uncharacterized protein kinases and also to investigate if the sequence between subdomains VII and VIII plays a role in substrate recognition or catalytic reaction.

It is important to emphasize that our phylogenetic analysis has limitations imposed by the fact we are dealing with an EST databank. For example, many putative sugarcane protein kinases were excluded from our analysis since the available sequences do not present most of the *pkinase *subdomains. Even though, the groups obtained are in good agreement with the classes, groups and families of plant protein kinases previously defined by the PlantsP database, despite differences in their classification methodology [122].

## Conclusion

In this work, the expression of 1,545 sugarcane SAS (mostly related to signal transduction components) was evaluated by cDNA microarrays in plants submitted to a variety of challenges: drought, phosphate starvation, herbivory by *Diatraea saccharalis *and endophytic bacteria inoculation (*Herbaspirillum seropedicae*/*Herbaspirillum rubrisubalbicans *and *Gluconacetobacter diazotrophicus*). Additionally, plants were treated with the phytohormones ABA and MeJA, important players in the responses to biotic and abiotic stresses.

To our knowledge, this is the first broad evaluation of sugarcane gene expression in response to biotic, abiotic and hormone inputs. Since ABA and MeJA play main roles in plant responses to a plethora of stimuli the analysis of genes regulated by these hormones is helpful in deciphering sugarcane defense pathways activated in response to stresses. Many of the differentially expressed genes belong to protein families described in the literature as associated to some of the processes studied, indicating that sugarcane responses are similar to those of other well-known plants, such as rice, maize and *Arabidopsis*. Additionally, functions were associated to genes poorly studied or novel genes such as genes with no hits in the public databases, genes encoding unknown proteins and undefined kinases/RLKs. The information generated by the protein kinase categorization using a phylogenetic approach, associated to the expression data obtained from microarray experiments, represents a useful tool in guiding the future characterization of these proteins.

Understanding the molecular mechanisms behind sugarcane stress responses will be useful for the improvement of sugarcane yield by genetic manipulation. This knowledge, allied to the use of genetic engineering, will potentially enable the development of sugarcane varieties tolerant to adverse conditions, such as drought and nutritional deficiency. Furthermore, the genes may be explored as molecular markers in traditional breeding programs or have their promoters cloned to accomplish transgene expression activated solely by a specific stimulus. It is important to emphasize the limitations intrinsic to the nature of the data presented. First, changes in mRNA levels do not always correlate to protein levels. Second, in the field, plants are exposed to a diversity of stressful conditions and the responses achieved by this combination of stimuli probably are not the same as the ones triggered by each individual stimulus. Nonetheless, the data generated in controlled experiments certainly represent an important step in the exploration of specific responses. The data also points candidates for gene silencing or overexpression experiments that may corroborate the hypothesis raised. With this in mind an expression panel is currently being constructed for several additional sugarcane cultivars tolerant or more susceptible to the stimuli that certainly will be valuable in guiding the selection of target genes. It will be important to expand the present studies to additional genotypes also if one wishes to compare the responses elicited by the different stimuli. The data obtained may reflect cultivar specific responses in the case of drought and endophytic bacteria interaction, since different cultivars were used in these experiments. The extent of genotypic variation among commercial cultivars is currently unknown. Evaluation of sugarcane responses in additional genotypes is underway to further validate commonly regulated pathways.

A databank was created that provides public access to the data described in this work, associated to tissue expression profiling and the SUCAST gene categories. As the SUCAST Project is an ongoing effort that aims to identify sugarcane signaling components and define their role in grasses, the database is expected to be updated each time new expression data from experiments with the SUCAST arrays are available. We expect the SUCAST database to become a useful tool for sugarcane transcriptome data mining and in guiding the selection of target genes to be modified in sugarcane and other grasses.

## Methods

### Plant material and cultivation

The cultivar SP90-1638 (Internal Technical Report, CTC, 2002), sensitive to drought, was used for the water deprivation experiments. The cultivar SP80-3280 sensitive to herbivory by *Diatraea saccharalis *[123] was adopted for the herbivory experiments. The same cultivar was used for phosphate deficiency and phytohormone treatment experiments. The cultivar SP70-1143 with high inputs of nitrogen obtained from BNF and with efficient association with endophytic bacteria [124] was used for inoculation with *Gluconacetobacter *and *Herbaspirillum*.

Sugarcane plantlets obtained from one-eyed seed sets were used for methyljasmonate treatment, water stress and herbivory experiments. For methyljasmonate treatments, one-eyed seed sets were planted in 200 ml plastic cups containing a commercial planting mix (Plantmax, Eucatex) for 20 days under greenhouse conditions and subsequently transferred to a growth chamber at 26°C on a 16 h/8 h light/dark cycle with a photon flux density of 70 μE.m^-2^.s^-1 ^to acclimate for 48 h. For the insect attack assays, one-eyed seed sets were planted in 200 ml plastic cups as described above and maintained in the greenhouse for 60 days when they were transferred to a growth chamber at 28°C on a 14 h/8 h light/dark cycle with a photon flux density of 70 μE.m^-2^.s^-1^. Sugarcane one-eyed seed sets were cultivated on moist sand for 15 days prior to drought experiments. For ABA treatment, plants derived from shoot apex of 2-month-old sugarcane plants were axenically *in vitro *cultivated for approximately three months in a growth chamber at 26°C on a 16 h/8 h light/dark cycle with a photon flux density of 70 μE.m^-2^.s^-1^. Sugarcane rooted plantlets obtained by sterile *in vitro *meristem culture and micropropagated according to the method of Hendre *et al*. [125] were used for nutritional deficiency and inoculation with endophytic bacteria experiments.

### Plant treatments

Plant treatments are described below. Three biological replicates were performed for each of these treatments. Two of the replicates were used for microarray experiments and one for real-time PCR reactions.

#### MeJA treatment

Plantlets were sprayed with a 100 μmol.L^-1 ^MeJA solution (Bedoukian Research Inc., Danbury, CT), whereas control plantlets were treated with distilled water. Leaves were collected 0, 1, 6 and 12 h after exposure to MeJA and immediately frozen in liquid nitrogen. Six plantlets were sampled for each time point.

#### ABA treatment

ABA (Sigma Chem. Co) was added to the culture medium to a final concentration of a 100 μmol.L^-1 ^whereas control plants were treated with distilled water. Leaves were collected 0, 0.5, 1, 6 and 12 h after exposure to ABA and immediately frozen in liquid nitrogen. Six plantlets were sampled for each time point.

#### Phosphate deficiency

Rooted plantlets were greenhouse acclimatized by initial cultivation on 1/20^th ^strength Hoagland and Arnon [126] nutrient solution. Nutrient solutions were replaced every 7 days increasing nutrient concentration to 1/4 strength in 3 weeks. Subsequently, plants were transferred to 2.8 L pots filled with fresh 1/4 strength nutrient solution. After one week, half of the plants were transferred to fresh solution containing 250 μM Pi, while the other half was transferred to nutrient solution deprived of phosphate (P_i_), with H_2_PO_4 _being replaced by H_2_SO_4 _[127]. Roots from each treatment (0 and 250 μM Pi) were harvested 6, 12, 24 and 48 h after the onset of phosphate starvation and immediately frozen in liquid nitrogen. For each time point, root samples of two plants were pooled.

#### Plant-N_2_-fixing endophytic bacteria association

Plantlets were inoculated as described [128] with 0.1 ml of a 10^6 ^10^7 ^cells/mL bacterial suspension. Controls were inoculated with medium only. The endophytic diazotrophic bacteria used were *Gluconacetobacter diazotrophicus *(PAL5 strain) or a mixture of *Herbaspirillum seropedicae *(HRC54 strain) and *H. rubrisubalbicans *(HCC103 strain). All plants were maintained at 30°C with a photon flux density of 60 μE.m^-2^.s^-1 ^for 12 h d^-1^. One day after the inoculation, plant tissues were examined for bacterial colonization by the Most Probable Number (MPN) estimation [129] and plantlets were collected and immediately frozen in liquid nitrogen. Five plantlets were pooled for each treatment.

#### Herbivory by Diatraea saccharalis

Sugarcane stalk borer larvae were grown on an artificial diet [130] and maintained at 25°C and 60 ± 10% relative humidity with a 14 h/10 h light/dark cycle. Second instar larvae were maintained under fasting conditions for 18 h prior to transfer. After transferring to plantlets, larvae were observed for a period of two hours to ensure complete boring into the sugarcane stalk. Control plantlets were kept unattacked. After 0.5 and 24 h of exposure to herbivory, plantlets were cut at the stalk/root zone and immediately frozen in liquid nitrogen. For each treatment, two plantlets were used for each time point.

#### Drought

The plants were transferred to pots containing moist sand, irrigated with Hoagland's solution [126] and maintained under greenhouse conditions. Regular watering was controlled using a Livingstone atmometer [131] and maintained for 90 days, being suppressed after this period for the experimental group. To control for water loss, soil samples were collected and the humid weight of each soil sample was compared with its dried weight, in order to verify the hydric loss in experimental plants. Aerial parts of the plants were collected 24, 72 and 120 h after the onset of drought for the control and experimental groups. Samples were collected and immediately frozen in liquid nitrogen. For each treatment, aerial parts of six plants were used for each time point.

#### RNA extraction

Frozen tissues were grinded using a homogenizer. Tissue samples of 2–2.5 g were weighted and grinded to a fine powder, in liquid nitrogen, using a pre-cooled mortar and pestle. The pulverized tissue was transferred to a 50 ml tube and homogenized with 5 ml Trizol (Invitrogen) per gram of tissue according to the manufacturer's instructions. RNA pellets were resuspended in 20 μl of warm diethyl pyrocarbonate-treated water, vortexing gently for about 15 min. RNA samples were quantified in a spectrophotometer and loaded on 1% agarose/formaldehyde gels for quality inspection.

### PCR amplification and array printing

cDNA microarray experiments were conducted essentially as reported previously [27]. Sugarcane cDNA plasmid clones obtained from the SUCEST collection were re-arranged and amplified in 100 μl PCR reactions (40 cycles, annealing at 51°C), directly from bacterial clones in culture, using T7 and SP6 primers. PCR products were purified by filtration using 96 well filter plates (Millipore Multiscreen MAFBN0B50). Samples were visualized on 1% agarose gels to inspect PCR-amplification quality and quantity. Purified PCR products (in 10 mM Tris-HCl solution at pH 8.0) were mixed with an equal volume of DMSO in 384 well V-bottom plates. Microarrays were constructed by arraying cDNA fragments on DMSO optimized metal-coated glass slides (type 7, GE Healthcare) using the Generation III Microarray Spotter (Molecular Dynamics). Each cDNA fragment was spotted on the slides at least twice (i.e., technical replicates). Following printing, the slides were allowed to dry and the spotted DNA was bound to the slides by UV-cross linking (50 mJ).

### Hybridization and selection of differentially expressed genes

Ten to fifteen micrograms of total RNA were reverse transcribed, labeled, and hybridized using the reagents provided with the CyScribe Post-Labeling kit (GE Healthcare) according to the manufacturer's instructions. The products of the labeling reactions were purified in Millipore Multiscreen filtering plates to remove unincorporated labeled nucleotides. Microarrays were co-hybridized with the fluorescently labeled probes. Hybridizations were performed overnight at 42°C in humid chambers. The slides were then washed in 1× SSC and 0.2% SDS (10 min, 55°C), twice in 0.1× SSC and 0.2% SDS (10 min, 55°C), and in 0.1 × SSC (1 min, RT). Slides were rinsed briefly in filtered milli-Q water and dried with a nitrogen stream. Each experimental step was carefully monitored to ensure high quality of the slides and extracted data. Slides were scanned using the Generation III Scanner (Molecular Dynamics) adjusting the photomultiplier tube (PMT) to 700 for both channels.

The microarray designed was composed of 1,830 genes selected from the SUCAST Catalogue. Hybridizations were carried out as depicted in Table 1. Two biological replicates were used for each microarray experiment. The 1,830 unique genes represented yielded 1,545 good-quality PCR fragments.

Images were processed and data collected using the ArrayVision (Imaging Research Inc.) software. Local median background was subtracted from the MTM (median-based trimmed mean) density for each spot [27]. Data from clones that generated poor-quality PCR fragments (no amplification or unspecific bands) or relative to saturated, low-intensity or poor-quality spots (visually inspected) were excluded.

The fluorescence ratios were visualized and normalized in the MxS space, where M is the base 2 logarithm of the intensities ratio and S is the base 2 logarithm of the average intensity of each spot. The M values were normalized to account for systematic errors using the LOWESS fitting [132]. The raw and normalized data are publicly available according to the MIAME guidelines at the GEO database under the accession numbers GSE4966 to GSE4971. Differentially expressed genes were defined as the extreme outliers in each experiment, using an intensity-dependent strategy modified from the HT-self method [133] and described in [34]. This method defines an intensity-dependent cutoff curve using the data from each hybridization, detecting non-parametrically genes with the greatest log-ratio changes (outliers) regardless of the absolute value of the log-ratio measurement. We defined as differentially expressed a gene that has at least 60% of their replicate-points above or below the cutoff curve in the two hybridizations of the biological *vs*. controls samples, indicating a reproducible result between the biological replicates. The number of technical replicates ranges from 2 to 16 since genes are spotted several times in the same array. The credibility level used to define outliers was 0.8.

### Clustering of expression data using Self Organizing Maps (SOM)

Biologically reproducible expression profiles were clustered with the Self Organizing Maps (SOM) method [36] using the Spotfire DecisionSite for Functional Genomics software (Spotfire, Somerville, Massachusetts) with default advanced parameters. For each experimental point, the median of the normalized M values among all technical replicates was calculated for each gene represented in the SUCAST microarray. The median values of M in each biological replicate were mean-centered in order to emphasize similarities in the deviations from the mean value by subtracting the average expression level of each gene along the time-course from the experimental measurement obtained in each experimental point. We considered an expression profile as biologically reproducible when the correlation coefficient was ≥ 0.7 between the mean-centered values from pair-wise biological replicate comparisons. SAS with at least one invalid M value (saturated, low-intensity or poor quality signals) were excluded from this analysis. The mean-centered values were averaged between the biological replicates and a Principal Component Analysis (PCA) was performed to estimate the number of groups to be generated by the SOM algorithm [134]. The results obtained are shown in additional file 11: Table 9, which demonstrate that the establishment of four to six groups for these data was enough to represent the main sources of variability among the selected patterns. The 2 × 2 and 2 × 3 geometries were tested when generating the SOM results for each of these treatments. We concluded that the 2 × 2 geometry in the case of MeJA treatment and a 2 × 3 geometry in the case of ABA treatment, phosphate starvation and drought resulted in groups with little internal variation.

### Validation of microarray results by real-time PCR (RT-PCR)

Two to five micrograms of total RNA (from a third biological replicate for each treatment) were treated with DNase (Invitrogen) according to the manufacturer's instructions and an aliquot of 7.5 μl of the treated RNA was reverse-transcribed using the SuperScript First-Strand Synthesis System for RT-PCR (Invitrogen). The 20 μl reverse transcription reactions contained the RNA template, 2 μl 10× RT buffer, 0.5 mM each dATP, dGTP, dCTP and dTTP, 50 ng random hexamers, 0.25 μg oligo(dT), 5 mM MgCl_2_, 10 mM DTT (dithiothreitol), 40 U RNase OUT and 50 U *SuperScript II Reverse Transcriptase*. RNA, random hexamers, dNTPs, and oligo(dT) were mixed first, incubated at 70°C for 5 min and placed on ice. The remaining components, except the *SuperScript II Reverse Transcriptase*, were added to the reaction, the mixture was heated to 25°C for 10 min and then incubated at 42°C for 2 min. The *SuperScript II Reverse Transcriptase *was added to each tube and the reaction was incubated at 42°C for 1.5 h, 72°C for 10 min, and chilled on ice. An identical reaction without the reverse transcriptase was performed as a control (no amplification control, NAC) to confirm the absence of genomic DNA. The cDNA product was treated with 2 U of RNaseH (Invitrogen) for 30 min at 37°C and for 10 min at 72°C. Real-time PCR reactions were performed using *SYBR Green PCR Master Mix *(Applied Biosystems) in a *GeneAmp 5700 Sequence Detection System *(Applied Biosystems). Primers were designed using the *Primer Express 2.0 *Software (Applied Biosystems). Real-time PCR reactions were performed in triplicates and contained 2 μl of a 1:10 dilution of the synthesized cDNA, primers to a final concentration of 600 nM each, 12.5 μl of the SYBR Green PCR Master Mix and PCR-grade water to a total volume of 25 μl. In the case of reactions using primers for 25S rRNA, a dilution of 1:1,000 of the synthesized cDNA was used. The parameters for the PCR reaction were 50°C for 2 min, 95°C for 10 min, 40 cycles of 95°C for 15 s and 60°C for 1 min. The specificity of the amplified products was evaluated by the analysis of the dissociation curves generated. No template controls (NTC) and no amplification controls (NAC) were run in order to confirm the absence of genomic DNA or reagent contamination. The relative expression ratio (experimental/control) was determined based on the 2^-ΔΔ*Ct *^method [135]. To access the statistical significance of expression ratios, we assumed a log-normal model and calculated the probability Pr(sample>reference) and Pr(sample < reference) for up- and down-regulated genes, respectively. The expression profile was considered validated when P ≥ 0.99. The primers used are shown in additional file 10: Table 8.

### Phylogenetic grouping of kinases

Sugarcane sequences containing a putative *pkinase *(catalytic domain of protein kinases) domain, as defined by the Pfam algorithm [43] were selected for the phylogenetic analysis of RLKs and other protein kinases. Protein kinase sequences from other organisms were retrieved in their majority from the PlantsP database [122] and used as drivers in the phylogenetic analysis. The sequences were aligned using ClustalW [136] with default parameters. The *pkinase *alignment was manually adjusted to preserve the conserved subdomains defined by Hanks and colleagues [118-120] for eukaryotic kinases using the Se-Al Sequence Alignment Editor [137]. The alignment was trimmed to remove gaps in most of the sequences. Sequences spanning less than 50% of this partial alignment were discarded. The alignment was analysed with PAUP [138] using the neighbor-joining (NJ) algorithm and distance as the criterion. Bootstrap analysis was conducted with 500 replicates using NJ/UPGMA and distance as the criterion.

## List of abbreviations used

ABA: abscisic acid, BNF: Biological Nitrogen Fixation, CDPKs: Calcium-Dependent Protein Kinases, GAPDH: glyceraldehyde 3-phosphate dehydrogenase, MeJA: methyl jasmonate, NJ: neighbor-joining, PCA: Principal Component Analysis, RLCKs: Receptor-Like Cytoplasmic Kinases, RLKs: Receptor-Like Kinases, SAS: Sugarcane Assembled Sequences, SOM: Self-Organizing Maps, SUCAST: The Sugarcane Signal Transduction Project, SUCAMET: Sugarcane Metabolism Project; SUCEST: The Sugarcane EST Project.

## Authors' contributions

GMS as the General Coordinator of the SUCEST Project, conceived, advised and coordinated most aspects of the study. GMS and FRR wrote the manuscript. FRR and FSPT produced the arrays, designed the experiments, performed the hybridizations and analysed the data. FRR also performed the real-time PCR experiments and the SOM clustering analysis. MYN and RZNV worked on the data statistical analysis and differential gene expression determination. FRR, FSPT and GSM annotated and catalogued the genes. GSM conceived the SUCEST-FUN, SUCAST and SUCAMET Databases. RV constructed the SUCEST-FUN database and MYN helped with computational issues. The following researchers were responsible for conducting plant experimentation as follows: phosphate starvation (RDCD, RSA, AVOF); sugarcane inoculation with endophytic bacteria (FV, ASH); herbivory attack (CB, AHM, MCSF); drought experiments (FAR, JAG, SMZ); phytohormone treatments (VER, MM). ECS was responsible for sugarcane cultivation at CTC, Piracicaba. All authors contributed, read, corrected and approved the final manuscript. The authors declare no conflict of interest in this work.

## Supplementary Material

Additional file 1 SUCAST SAS showing differential expression in response to the applied treatments. The table lists all SAS whose expression was enriched or decreased in both biological samples for each experimental point. The plus sign indicates that the SAS expression is up-regulated, the minus sign indicates that the gene expression is down-regulated. The asterisk indicates that the SAS identity was not confirmed by re-sequencing. The table also shows the expression profile of these genes in six sugarcane tissues (**revised from [27]). The last four columns indicate the SOM groups in which these SAS were included (numbered according to Figure 1). The expression data for sugarcane tissues were inferred from microarray hybridizations of tissue samples against a common reference constituted by an equimolar mixture of the tissues sampled [27]. FL = flowers, LB = lateral buds, LV = leaves, RT = roots, IN1 = first internodes, IN4 = fourth internodes, PD = phosphate deficiency, H = inoculation with *Herbaspirillum *spp., GD = inoculation with *Gluconacetobacter diazotrophicus*. Click here for file

Additional file 2 Log_2 _ratios of microarray signals between the Cy3 and Cy5 channels. For each clone, the median intensity value of the technical replicates was used to calculate the ratio between the experimental samples and the reference sample. The numbers 1 and 2 denote the different biological samples used for each experiment. The asterisk indicates that the SAS identity was not confirmed by re-sequencing. Only valid values are shown, excluding data from saturated, low-intensity and low-quality spots. Data from clones for which PCR reactions produced low yields or multiple bands were also removed. The table contains 1,555 elements and not 1,545. This is due to the fact that some SAS are represented more than once in the Table. This occurs when the same SAS is represented in more than one position in the 384-well plates and some of these positions did not have their identity validated by re-sequencing. In these cases, we opted to analyse these data separately. Click here for file

Additional file 3 SUCAST protein kinases showing differential expression in response to the applied conditions. For SAS included in the SOM clustering analysis, the group to which the SAS belongs is indicated. The plus sign indicates that the SAS expression is up-regulated, the minus sign indicates that the gene expression is down-regulated. The asterisk indicates that the SAS identity was not confirmed by re-sequencing. The last four columns indicate the SOM groups in which these SAS were included (numbered according to Figure 1). PD = phosphate deficiency, H = inoculation with *Herbaspirillum *spp., GD = inoculation with *Gluconacetobacter diazotrophicus*. Click here for file

Additional file 4 Components of the SOM groups generated for ABA treatment. The table indexes all genes that were included in the clustering analysis (correlation coefficient ≥ 0.7 between the expression profiles obtained for the two biological replicates of a particular experiment) and their respective groups. The asterisk indicates that the SAS identity was not confirmed by re-sequencing. Click here for file

Additional file 5 Components of the SOM groups generated for MeJA treatment. The table indexes all genes that were included in the clustering analysis (correlation coefficient ≥ 0.7 between the expression profiles obtained for the two biological replicates of a particular experiment) and their respective groups. The asterisk indicates that the SAS identity was not confirmed by re-sequencing. Click here for file

Additional file 6 Components of the SOM groups generated for phosphate deficiency treatment. The table indexes all genes that were included in the clustering analysis (correlation coefficient ≥ 0.7 between the expression profiles obtained for the two biological replicates of a particular experiment) and their respective groups. The asterisk indicates that the SAS identity was not confirmed by re-sequencing. Click here for file

Additional file 7 Components of the SOM groups generated for drought treatment. The table indexes all genes that were included in the clustering analysis (correlation coefficient ≥ 0.7 between the expression profiles obtained for the two biological replicates of a particular experiment) and their respective groups. The asterisk indicates that the SAS identity was not confirmed by re-sequencing. ** The two different results obtained for this SAS are related to different positions in the 384-well plates. In both positions the SAS identity was not validated by re-sequencing. Because of this, these positions were treated separately. Click here for file

Additional file 8 Neighbor-joining (NJ) tree of kinase domains from sugarcane protein kinases. Selected kinase domains were aligned to construct a distance tree with the NJ algorithm. Bootstrap values greater than 50% (500 replicates) are shown for nodes in the tree. The RLKs/RLCKs group is highlighted in gray and only some representatives of this group are included. The undefined kinases are highlighted in red. Drivers are in italic. The SAS name in the figure is preceded by a prefix based on the BLAST similarity searches as indicated in the Annotation Box. The branch color indicates the presence of additional Pfam [43] domain besides the pkinase domain as indicated in the Domains Box. Click here for file

Additional file 9 Neighbor-joining (NJ) tree of kinase domains of sugarcane RLKs and RLCKs. Selected kinase domains from sugarcane RLKs and RLCKs and from other plants (drivers) were aligned to construct a distance tree with the NJ algorithm. Bootstrap values greater than 50% (500 replicates) are shown for nodes in the tree. Drivers are in italic. Undefined RLKs and RLCKs are highlighted in red. The SAS name in the figure is preceded by a prefix based on the BLAST similarity searches as indicated in the Annotation Box. The branch color indicates the presence of additional Pfam [43] and SMART [139] domains besides the pkinase domain as indicated in the Domains Box. Click here for file

Additional file 10 Sugarcane genes and primers selected for real-time PCR experiments. Primers were designed using the Primer Express 2.0 Software (Applied Biosystems) and BLAST searches against the SUCEST database were conducted to ensure the specificity of the selected primers. The column Experimental Datapoints lists the samples analysed. Primer sequences for endogenous references were retrieved from [27] and [38]. The *P value *is the probability Pr(sample>reference) and Pr(sample < reference) for up- and down-regulated genes, respectively. The expression profile was considered validated when *P *≥ 0.99. Click here for file

Additional file 11 PCA analysis for defining the number of groups in SOM. The difference between the magnitude of principal component eigenvalues for each treatment (columns) estimates how many groups are minimally necessary in each SOM grouping analysis. When the difference is near zero, there is no substantial information added by increasing the number of seed groups in SOM. Click here for file

## Figures and Tables

**Table 1:** 

**MeJA**			**ABA**			**Herbivory**			**Phosphate starvation**			***Gluconacetobacter***			***Herbaspirillum***			**Drought**		
**Cy3**		**Cy5**	**Cy3**		**Cy5**	**Cy3**		**Cy5**	**Cy3**		**Cy5**	**Cy3**		**Cy5**	**Cy3**		**Cy5**	**Cy3**		**Cy5**

0h (C1)	vs.	1h (E1)	0h (C1)	vs.	30min (E1)	30min (E1)	vs.	30min (C1)	6h (E1)	vs.	6h (C1)	E1	vs.	C1	E1	vs.	C1	24h (C1)	vs.	24 h (E1)
0h (C1)	vs.	6h (E1)	0h (C1)	vs.	1h (E1)	24h (E1)	vs.	24h (C1)	12h (E1)	vs.	12h (C1)	C2	vs.	E2	C2	vs.	E2	72h (C1)	vs.	72 h (E1)
0h (C1)	vs.	12h (E1)	0h (C1)	vs.	6h (E1)	30min (C2)	vs.	30min (E2)	24h (E1)	vs.	24h (C1)							120h (C1)	vs.	120 h (E1)
1h (E2)	vs.	0h (C2)	0h (C1)	vs.	12h (E1)	24h (C2)	vs.	24h (E2)	48h (E1)	vs.	48h (C1)							24 h (E2)	vs.	24h (C2)
6h (E2)	vs.	0h (C2)	30min (E2)	vs.	0h (C2)				6h (C2)	vs.	6h (E2)							72 h (E2)	vs.	72h (C2)
12h (E2)	vs.	0h (C2)	1h (E2)	vs.	0h (C2)				12h (C2)	vs.	12h (E2)							120 h (E2)	vs.	120h (C2)
			6h (E2)	vs.	0h (C2)				24h (C2)	vs.	24h (E2)									
			12h (E2)	vs.	0h (C2)				48h (C2)	vs.	48h (E2)									

**SP80-3280**												**SP70-1143**						**SP90-1638**		

cDNA microarray hybridizations. The table indicates which CyDye was used to label each sample and the experimental design. Two biological replicates were sampled for each treatment (E1 and E2) or control (C1 and C2) experiments. The table also indicates the cultivar used in each experiment.
